# Supplementary figures and images for: Optimum time for hand pollination in yam (Dioscorea spp.)
Source: PLoS One. 2022 Aug 18;17(8):e0269670. doi: 10.1371/journal.pone.0269670 (PMC9387836; doi:10.1371/journal.pone.0269670)

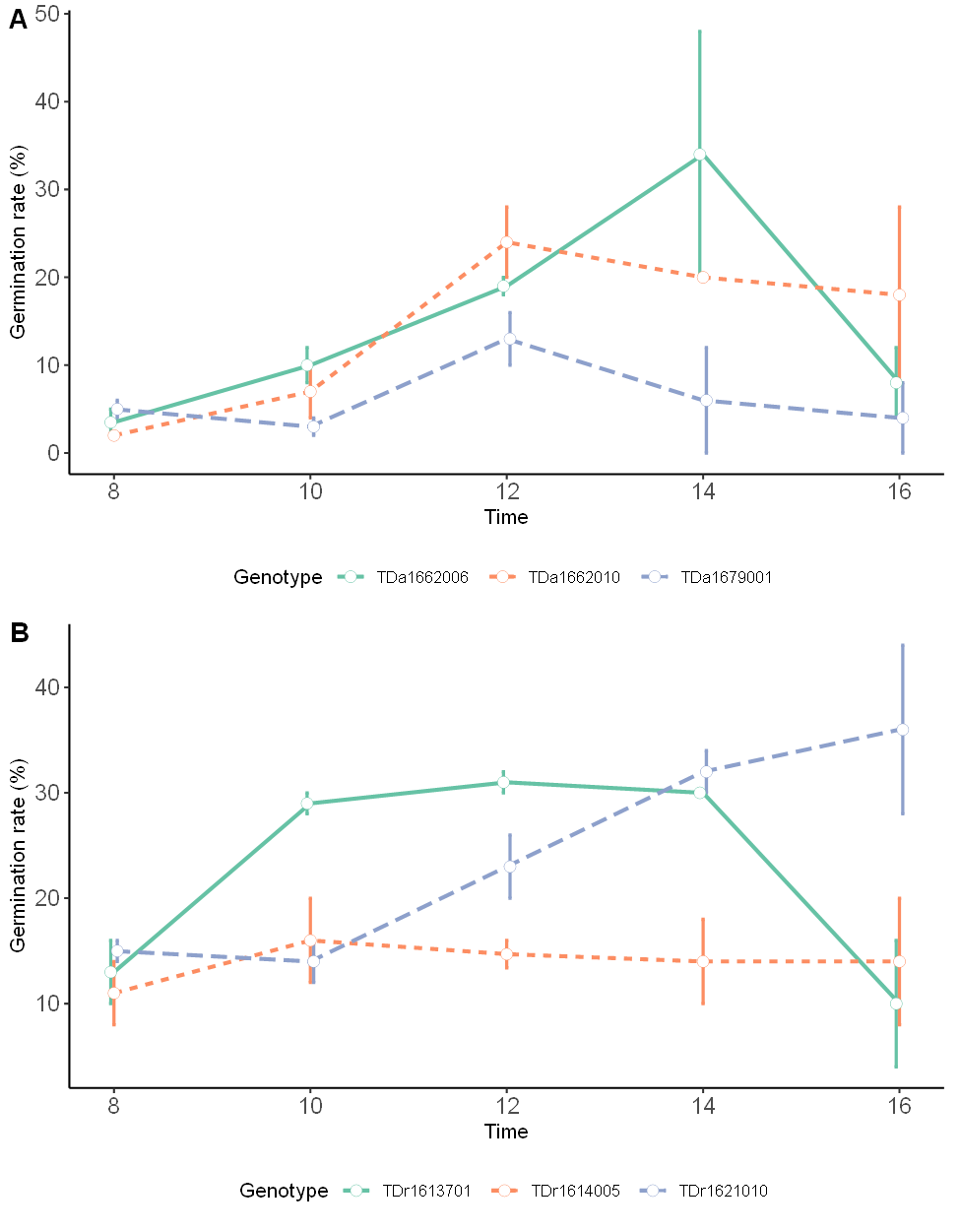


**S1 Fig. Genotypic variability in pollen germination in *D. alata* (A) and *D. rotundata* (B).**

Supplement: S1 Fig — (DOCX) [file pone.0269670.s001.docx]
