# Supplementary material for: Optimum time for hand pollination in yam (Dioscorea spp.)
Source: PLoS One. 2022 Aug 18;17(8):e0269670. doi: 10.1371/journal.pone.0269670 (PMC9387836; doi:10.1371/journal.pone.0269670)
Supplement: S2 Fig — (DOCX) [file pone.0269670.s002.docx]

**S2 Fig. Pollination success rates across crossing weeks for 2020 and 2021. (A) *D. alata* and (B) *D. rotundata.*** Week 1 for *D. alata* corresponded to the second week of October while week 9 was the second week of December. Week 1 for *D. rotundata* referred to second week of August while week 9 corresponded to the second week of October.
