# Supplementary material for: Optimum time for hand pollination in yam (Dioscorea spp.)
Source: PLoS One. 2022 Aug 18;17(8):e0269670. doi: 10.1371/journal.pone.0269670 (PMC9387836; doi:10.1371/journal.pone.0269670)
Supplement: S3 Fig — (DOCX) [file pone.0269670.s003.docx]

**S3 Fig. Dynamics of SPE (A), SSR (B) and SV (C) regardless of the species**
