# Supplementary material for: Optimum time for hand pollination in yam (Dioscorea spp.)
Source: PLoS One. 2022 Aug 18;17(8):e0269670. doi: 10.1371/journal.pone.0269670 (PMC9387836; doi:10.1371/journal.pone.0269670)
Supplement: S7 Fig — (DOCX) [file pone.0269670.s007.docx]

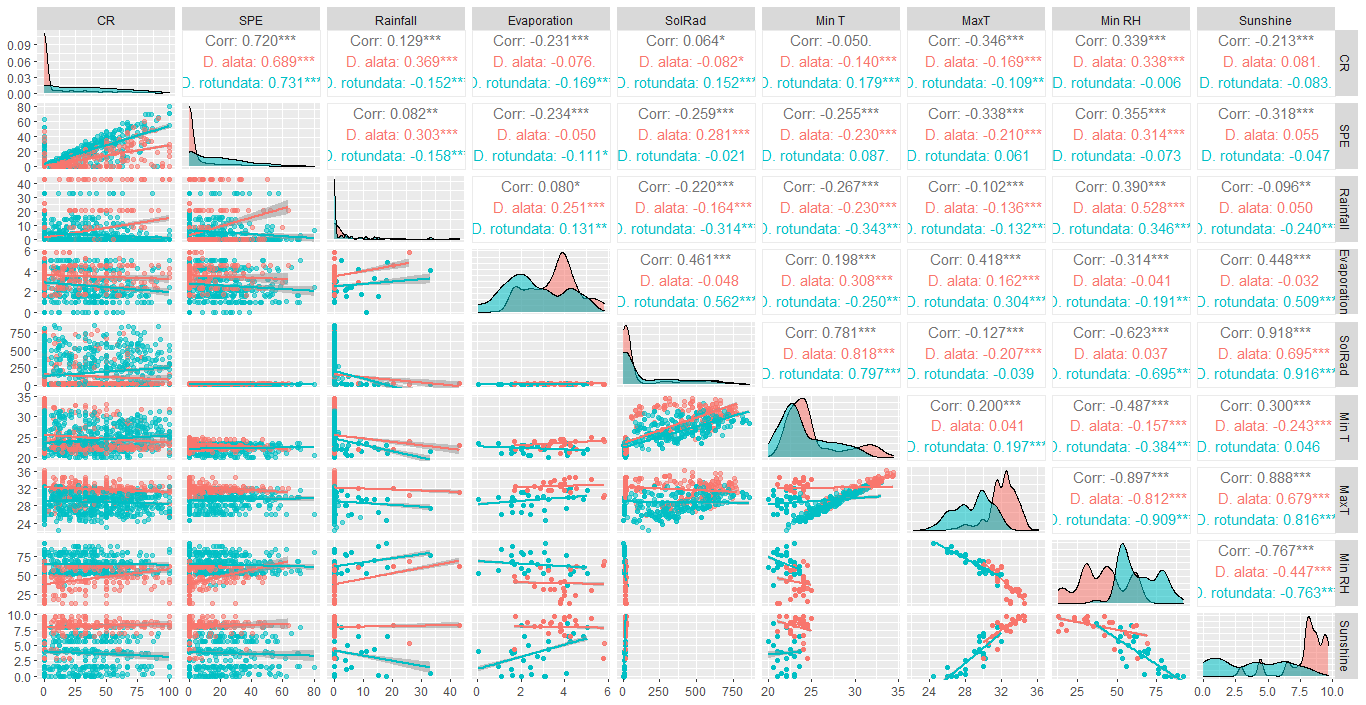


**S7 Fig. Relationship among pollination success related traits and the weather parameters using the species as a factor.** CR=crossability rate/pollination success, SPE = seed production efficiency, SolRad = solar radiation, Min T = minimum temperature, MaxT = maximum temperature, Min RH = minimum relative humidity.
