# Supplementary material for: Optimum time for hand pollination in yam (Dioscorea spp.)
Source: PLoS One. 2022 Aug 18;17(8):e0269670. doi: 10.1371/journal.pone.0269670 (PMC9387836; doi:10.1371/journal.pone.0269670)
Supplement: S8 Fig — (DOCX) [file pone.0269670.s008.docx]

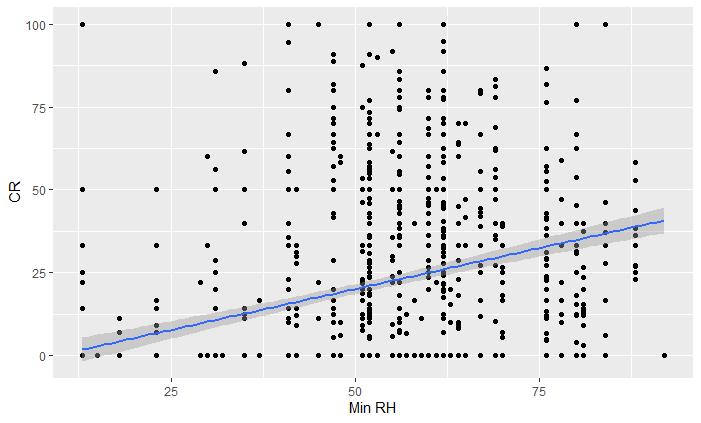

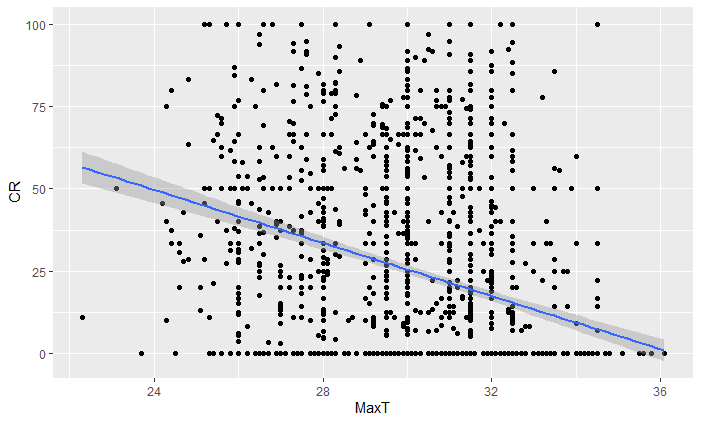


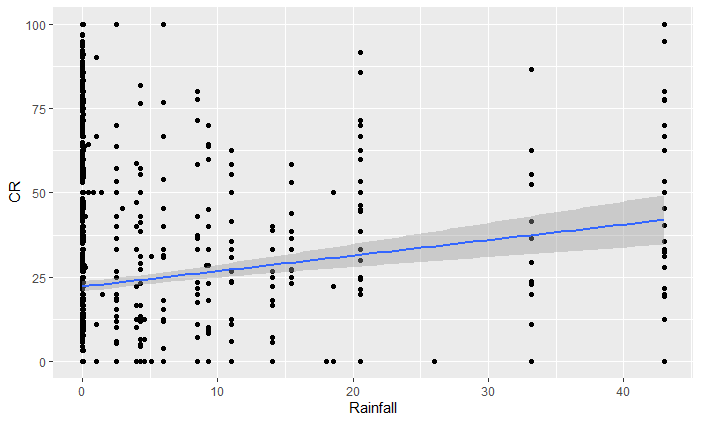

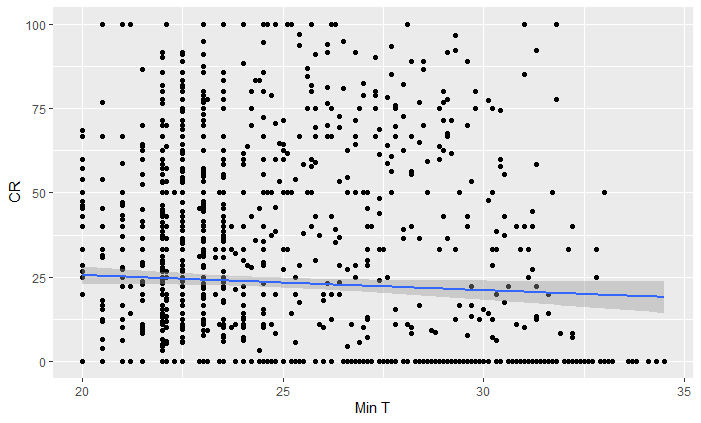


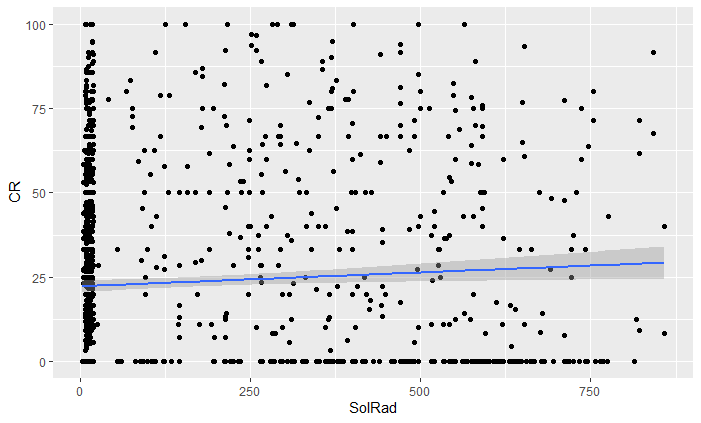

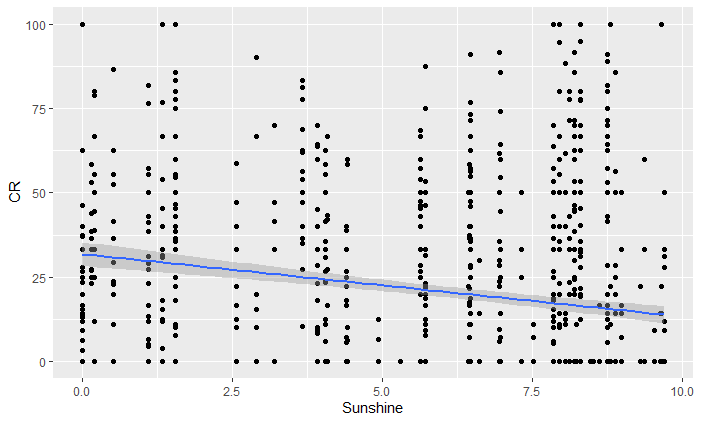


**S8 Fig. Weather variables having the most effect on yam pollination success regardless of the species.** CR=crossability rate/pollination success, SolRad = solar radiation, Min T = minimum temperature, MaxT = maximum temperature, Min RH = minimum relative humidity.
