# Supplementary material for: Optimum time for hand pollination in yam (Dioscorea spp.)
Source: PLoS One. 2022 Aug 18;17(8):e0269670. doi: 10.1371/journal.pone.0269670 (PMC9387836; doi:10.1371/journal.pone.0269670)
Supplement: S9 Fig — (DOCX) [file pone.0269670.s009.docx]

**
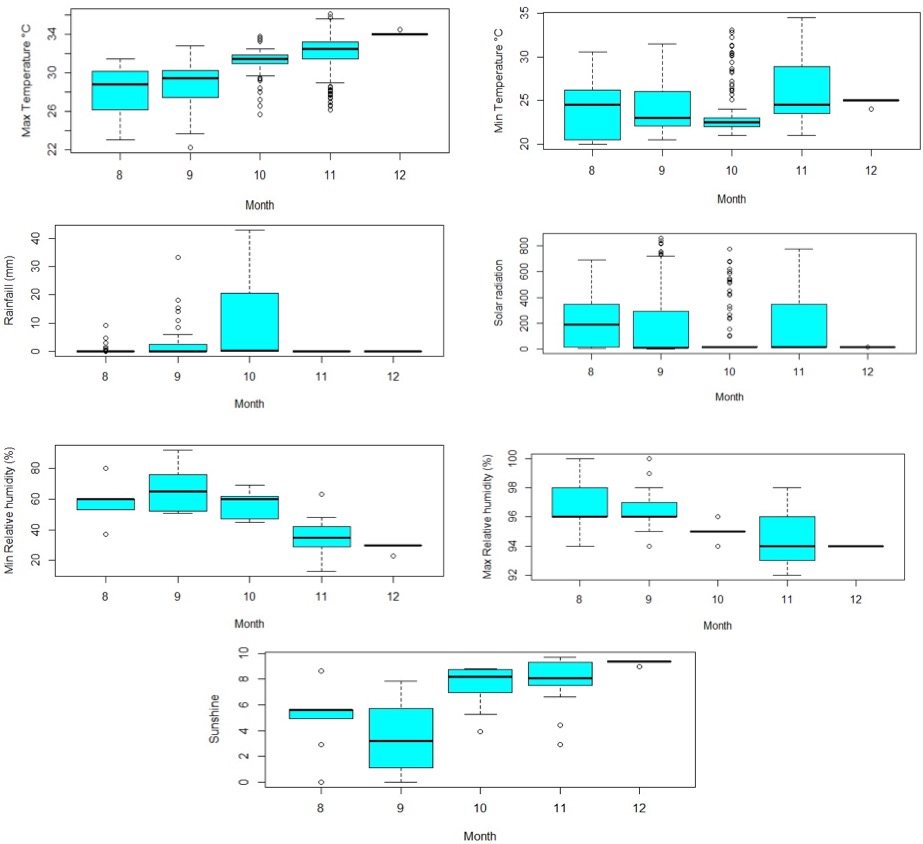
**

**S9 Fig. Fluctuations in weather data across months covering the yam flowering window.** 8 refers to August and 12 to December.
