# Supplementary material for: Optimum time for hand pollination in yam (Dioscorea spp.)
Source: PLoS One. 2022 Aug 18;17(8):e0269670. doi: 10.1371/journal.pone.0269670 (PMC9387836; doi:10.1371/journal.pone.0269670)
Supplement: S1 Table — (DOCX) [file pone.0269670.s010.docx]

**S1 Table. Description of yam genotypes used in hand pollination experiment**

| **Species** | **Genotype** | **Sex** | **Type** |
| --- | --- | --- | --- |
| *D. rotundata* | TDr1615003 | Female | Breeding line |
|  | TDr2965A | Female | Breeding line |
|  | TDr16010008 | Female | Breeding line |
|  | TDr1613701 | Male | Breeding line |
|  | TDr1542068 | Male | Breeding line |
|  | TDr1621010 | Male | Breeding line |
| *D. alata* | TDa160303 | Female | Breeding line |
|  | TDa1662002 | Female | Breeding line |
|  | TDa160402 | Female | Breeding line |
|  | TDa1662006 | Male | Breeding line |
|  | TDa1662010 | Male | Breeding line |
|  | TDa1679001 | Male | Breeding line |
