# Supplementary material for: Optimum time for hand pollination in yam (Dioscorea spp.)
Source: PLoS One. 2022 Aug 18;17(8):e0269670. doi: 10.1371/journal.pone.0269670 (PMC9387836; doi:10.1371/journal.pone.0269670)
Supplement: S2 Table — (DOCX) [file pone.0269670.s011.docx]

**S2 Table. ANOVA table for pollination success across crossing hours for *D. alata***

| **Sources of variation** | **DF** | **SS** | **MS** | **F value** | **P-value** |
| --- | --- | --- | --- | --- | --- |
| Time (T) | 8 | 35593 | 4449 | 8.061 | 9.41e-11*** |
| Genotype (G) | 8 | 258052 | 32256 | 58.445 | < 2e-16*** |
| T × G | 56 | 29429 | 526 | 0.952 | 0.577 ns |
| Residuals | 1926 | 1062981 | 552 |  |  |

DF = degrees of freedom, SS = sum of squares, MS = mean squares, ns = non-significant at p =0.05, *** = very highly significant at p = 0.05.
