# Supplementary material for: Optimum time for hand pollination in yam (Dioscorea spp.)
Source: PLoS One. 2022 Aug 18;17(8):e0269670. doi: 10.1371/journal.pone.0269670 (PMC9387836; doi:10.1371/journal.pone.0269670)
Supplement: S3 Table — (DOCX) [file pone.0269670.s012.docx]

**S3 Table. Comparison of weather data for 2020 and 2021 yam crossing windows**

|  | **2021** | | | | | **2020** | | | | |
| --- | --- | --- | --- | --- | --- | --- | --- | --- | --- | --- |
| **Parameters** | **Mean** | **SE** | **Min** | **Max** | **Sum** | **Mean** | **SE** | **Min** | **Max** | **Sum** |
| Evaporation (mm) | 3.11 | 0.042 | 0.08 | 5.78 | 2971.59 | 2.82 | 0.205 | 0 | 43 | 4243.5 |
| Wind speed (km h^-1^) | 2.98 | 0.065 | 0 | 8.682 | 4473.37 | 3.10 | 0.042 | 0.08 | 5.78 | 2971.59 |
| Solar radiation (MJ m^−2^ day^−1^) | 152.51 | 5.76 | 3.57 | 858.16 | 228774.3 | 14.1 | 0.065 | 3.60 | 19.4 | 13900.43 |
| Minimum Temperature (°C) | 24.68 | 0.081 | 20 | 34.5 | 37020.5 | 22.83 | 2.761 | 20.00 | 25.00 | 22492.00 |
| Maximum Temperature (°C) | 30.51 | 0.063 | 22.3 | 36.1 | 45775.5 | 31.03 | 2.081 | 24.50 | 34.5 | 5967.17 |
| Minimum relative humidity (%) | 50.86 | 0.57 | 13 | 92 | 50102 | 50.86 | 25.063 | 22.30 | 92.00 | 50102 |
| Maximum relative humidity (%) | 95.47 | 0.054 | 92 | 100 | 94043 | 95.47 | 0.572 | 92.00 | 100 | 94043 |
| Sunshine (h) | 6.076 | 0.096 | 0 | 9.7 | 5967.17 | 6.08 | 0.054 | 0 | 9.7 | 5967.17 |
